# Supplementary material for: Are carbon emissions trading and green financial instruments synergistic? -Comprehensive quantitative research based on content analysis
Source: PLoS One. 2024 Mar 7;19(3):e0298601. doi: 10.1371/journal.pone.0298601 (PMC10919601; doi:10.1371/journal.pone.0298601)
Supplement: S1 Data — (ZIP) [file pone.0298601.s001.zip › Data/地级市Z_F工作报告.zip!/╣π╢1⁄2/╔╟╬▓╩╨/└┤╫╘╣1⁄2╓┌║┼í╛┬φ┐╦╩2╛▌═°í┐_2012.docx]

各位代表：

　　我代表市人民政府向大会作政府工作报告，请予审议，并请政协各位委员和其他列席人员提出意见。

五年工作回顾

　　过去五年是极不平凡的五年，是我市经济社会发展最快、城乡面貌变化最大、人民得到实惠最多的五年。五年来，在省委、省政府及市委的正确领导下，在市人大和市政协的监督支持下，市政府坚持以科学发展观统揽全局，认真落实党中央、国务院和省委、省政府各项决策部署，以"五年大变化"和全面砍掉落后"尾巴"为目标，大力推进"三基地"、"三经济带"、"八大工程"建设，顺利完成了市五届人大一次会议确定的目标任务。

　　一、综合实力不断增强

　　经济总量五年翻番。2011年实现地区生产总值550亿元（预计数，下同），比2006年翻了一番多，年均增长16.7%，比建市以来年均增长速度快2.7个百分点，经济发展处于历史最好时期。

　　发展质量不断提高。2011年全市财政一般预算收入32.45亿元，是2006年的3.5倍，年均增长28.5％；全市金融机构年末本外币存款余额352亿元，贷款余额195亿元，分别是2006年末的2.1倍和2.4倍。市区居民人均可支配收入15585元，年均增长12.1%；农民人均纯收入7250元，年均增长12.6%。

　　扩大内需成效明显。坚持以"四推进一保持"为总揽，应对国际金融危机，促进经济增长。2011年全市完成固定资产投资326亿元，是2006年的3.5倍，年均增长28.6%；社会消费品零售总额415亿元，是2006年的2.7倍，年均增长21.9%。

　　对外开放不断扩大。外源经济稳步增长，五年招商引资共签约400多项，合同投资总额3000亿元。2011年全市外贸出口总额13.1亿美元，是2006年的1.5倍，年均增长8.9%；进出口货运量260万吨，是2006年的5.5倍，年均增长40.5%；实际利用外资3.4亿美元，是2006年的2.5倍，年均增长20.1%。

　　二、产业发展态势良好

　　产业集聚效应日益凸显，科技创新能力持续增强，产业结构不断优化，三次产业结构由2006年的20.6︰43.2︰36.2调整为2011年的16.3︰46.1︰37.6，形成以工业为主导、三次产业协调发展的格局。

　　工业体系初步建立。基本形成以电力能源、电子信息、石油化工、宝石加工、金银首饰、纺织服装、食品制造等产业共同发展的工业体系。2011年全市完成规模以上工业增加值140亿元，年均增长29%；高技术产业产值105亿元，年均增长14.7%，电子信息产业成为支柱产业，全市拥有电子信息企业20多家，完成工业产值占规模以上工业产值比重的两成；信利、五丰水产等5个项目列入省现代产业500强。电力能源基地初具规模。汕尾电厂四台机组建成投产，全市电源装机容量达到276万千瓦，陆丰核电、华润海丰电厂等重点项目获国家核准。

　　特色农业成效显著。绿色产业经济带和蓝色产业经济带初步形成。全市拥有省级现代农业示范园区11个；农业龙头企业57家，其中国家级1家，省级6家；农民专业合作组织196家。农机综合水平达到50%。农业品牌建设实现新突破，2011年全市11个农产品被评为省名牌产品，我市首次评选出10大金牌农产品和20大名优农产品。农产品抽检合格率达到98%。海洋渔业基础设施不断完善，汕尾渔港被评为全国"文明渔港"。汕尾已成为珠三角地区农产品供应基地。

　　服务业全面发展。"万村千乡"市场建设工程扎实推进，城乡购物环境明显改善，商贸流通畅旺有序。2009年以来，全市共销售家电下乡产品5万多台（件），实现销售金额1.2亿元。旅游业蓬勃发展，2011年全市旅游总收入59亿元，是2006年的3.8倍。房地产业、交通运输、邮电通信业持续快速发展。2011年完成第三产业增加值207亿元，是2006年的2.2倍。

　　产业园区建设取得突破。深汕特别合作区列入省"十二五"规划，上升为省级发展战略。基础配套项目建设加快推进，完成投资10亿元。招商引资成果丰硕，腾讯、恒兴水产、艾特航模、芯灵电子等21家企业落户合作区。在第四届侨博会上，我市经贸活动，共签约、开工、竣工项目139个，投资总规模1724亿元。其中深汕特别合作区签约项目20个，总投资130亿元；开工项目8个，总投资156亿元；剪彩项目7个，总投资38亿元。

　　自主创新能力明显增强。谋求后发先转型，加快产业集聚，形成省级专业镇6个，市级专业镇12个，海丰敏兴、城区万盛等毛纺企业已拥有6000多台数控织机。通过内生、外引、合作等多方努力，推动以企业为主体的自主创新。五年全市累计专利授权量1322项，年均增长20%，突破性获得4项省级科技进步奖、2项省专利优秀奖；建立了"广东省液晶及有机显示工程技术研究开发中心"。

　　三、宜居建设加快推进

　　积极实施"宜居城乡"发展战略，城市基础设施建设加快推进，城乡面貌发生喜人变化。2010年被评为"最喜爱休闲城市"，2011年上榜"中国最具魅力城市"。

　　城乡规划科学有序。围绕打造"一（中）心一轴、一带三片（区）、多组团"城市空间发展结构，完成市区规划修编，实现城市总体规划、产业布局、土地利用、环境保护"四规合一"；全市已有295个村庄完成编制规划，基本形成多层级、广覆盖的城乡规划体系。

　　交通网络不断完善。五年投入公路建设28亿元，新（改）建公路1608公里，国道324线和行政村公路硬底化改造任务全面完成；厦深铁路汕尾段开工建设，成为广东境内全线建设进度最快的路段；潮惠高速公路汕尾段顺利动工；广州至汕尾高铁、龙川至汕尾铁路、兴宁至汕尾高速公路前期工作加快推进。市政道路建设成效显著，汕遮路、海汕路西闸至埔边段（改建）、金湖路、腾飞路东段、成业路南段等一批市政主干道建成通车。汕尾红海湾港区获国务院批准对外开放，并通过省级验收。

　　城乡建设加快推进。电网建设、城市燃气管道、供水管网等配套设施不断完善，品清湖和坎下城综合治理取得突破，碧桂园等大型房地产项目开盘，城市整体形象明显提升。城镇化水平达到54.2%，中心市区面积由建市初的4平方公里扩展到26平方公里。"三旧"改造初见成效，陆河生态文明县试点建设扎实推进，城乡农田水利基础设施进一步完善，"三防"能力不断提升。

　　生态环境更加优美。修堤、减排、治污、绿化、改水等"生态安全五大工程"深入实施，森林覆盖率达到49.2%，城市人均公共绿地9.51平方米，完成79宗病险水库除险加固工程和88公里 "百里海堤爱海工程"建设。实现污染减排"砍尾"目标，完成省下达我市的单位GDP能耗和减排任务。污水处理厂、市生活垃圾无害化处理中心建设加快推进。

　　四、社会事业全面发展

　　社会保障取得新突破。社会保险实现市级统筹，新型农村、城镇居民养老保险试点及全民医保工作顺利推进，城乡低保、五保供养基本实现应保尽保。全市基本养老保险覆盖率67%，基本医疗保险覆盖率92%，新农合覆盖率达到100%。企业离退休人员养老金比2006年底增加1倍。社会救助体系不断完善，社会福利和慈善事业进一步发展。五年建成保障性住房3076套，完成农村饮水安全工程60宗，惠及农村人口102万人；改造农村危房18207户，农村"一保五难"问题基本解决。五年全市城镇新增就业20.2万人，培训农村劳动力10.7万人，转移就业24万人；2011年城镇登记失业率为2.7%。扶贫"双到"工作成效明显。2010年以来，全市投入扶贫资金7.1亿元，实施帮扶项目992个，村集体经济收入平均增收1.3万元，贫困户人均增收526元。

　　教育事业取得新突破。全面实现九年免费义务教育。普及高中阶段教育办学规模实现翻一番，高中阶段毛入学率从2006年的37.45%增至2011年的85.76%，提高48个百分点，顺利通过省普高考核验收。全面解决代课教师问题。中职教育不断扩张，普职协调发展，普职比由2007年的7︰3优化到2011年的4.8︰5.2，实现了普职在校生比例大体相当目标。"扶贫济困"活动募集教育发展资金13.8亿元。规划建设汕尾教育园区，华师附中汕尾学校完成首期建设并招生，陆河教育园区大部分项目已交付使用。教育教学质量稳步提高，2011年本科入围人数首次突破5000人大关；成功摘除省义务教育防辍重点管理地区的"帽子"。

　　卫生事业取得新突破。医药卫生体制改革不断深化，启动公立医院改革，全面实施基本药物制度。改造乡镇卫生院41家，市第一人民医院综合大楼和中医院新院建设进展顺利，基层医疗卫生服务覆盖率达到100%，逸挥基金医院成功创建三级综合性医院。

　　文化事业取得新突破。文化惠民工程加快推进，覆盖城乡的公共文化服务设施网络基本形成。群众性文化活动蓬勃开展，成功举办亚运会火炬传递文艺表演、庆祝建党90周年大型文艺晚会等活动；文艺创作活跃，先后获得国家级文艺奖项20多件，省级奖项150多件；我市被授予"中国民间文化艺术之乡"，海丰县被评为南粤锦绣工程文化先进县，碣石镇被评为国家级历史文化名镇，麒麟舞、河田高景和甲子英歌舞入选第二批、第三批国家非物质文化遗产名录；第一部西秦原创戏《留取丹心照汗青》获省艺术节7项奖；陆丰滚地金龙获第十届中国民间文艺山花奖，实现了我市国家级最高奖零的突破。

　　平安建设取得新突破。大力开展大平安工程建设，信访工作机制不断创新，综治信访维稳中心建设加快推进，全市54个镇（街道）和860个村（居）委三级平台高效运行，社会矛盾得到及时有效化解，刑事立案数、每万人刑事发案数均排在全省末位。应急管理工作不断加强，安全生产形势总体平稳。打击非法传销成效显著。走私贩私、制假售假等非法活动得到遏制。产品质量和食品药品安全专项整治成效明显。城市管理水平不断提高，全市有96个社区达到省"六好"平安和谐社区建设标准。

　　社会事业全面进步。成功举办第七届世界惠州同乡恳亲会、第四届侨博会暨深汕特别合作区招商会、第14届亚洲帆船锦标赛，协办第16届亚运会帆船赛。全民健身运动蓬勃开展，竞技体育水平稳步提高。五年累计获得省级以上竞技体育比赛奖牌88.5枚，市体育馆、农村农民健身工程等建成投入使用。人口计生工作取得新成绩，全市计划生育率97.55%，市城区、海丰由"二类"升"一类"，陆河、陆丰由"三类"升"二类"。顺利完成第二次经济普查和第六次人口普查工作。连续两次被评为"双拥模范城"。

粮食、物价、统计、气象、地震、移民、口岸、妇女儿童、工会、青年、外事侨务、港澳台事务等工作取得新成效。民族宗教、国防、林纸、司法、法制、人防、消防、档案修志、仲裁、老龄、残疾人等工作取得新进展。

　　五、民主法制与政府自身建设不断加强

　　依法行政、高效行政、政府改革、廉政建设、政务公开全面推进。自觉接受人大的法律监督和政协的民主监督，定期向市人大报告、向市政协通报工作。五年共办理人大代表议案6件、建议100件，政协提案390件，办复率100%。法治城市创建活动深入开展，全面完成"五五"普法。行政管理、投资、财税、交通、粮食流通、科技、教育、文化、卫生、城市公用事业等体制改革有序推进，政府机构、富县强镇事权、事业单位人事、林权制度等重点领域改革取得突破。审计、监督工作力度加大，"责任、督查、奖罚"三大机制全面落实，执行力不断提高。专家咨询论证、社会公示等制度进一步完善，民主评议公务、网络问政、"行风热线"形成常态化机制，损害群众利益的不正之风得到遏制，机关作风明显转变，办事效率和服务水平有所提高。

　　各位代表，过去五年取得的成绩，是在省委、省政府及市委的正确领导下，在市人大及其常委会、市政协的大力支持下，全市人民团结奋斗的结果。在此，我谨代表市人民政府，向关心支持汕尾发展、倾注心血、付出辛勤劳动的各界人士和广大市民表示崇高的敬意和衷心的感谢！

　　回顾过去五年的工作，我们深深体会到：必须坚持解放思想、先行先试，善于把汕尾工作放在全省改革发展稳定大局中去思考谋划，敢于摆脱传统的思维束缚和模式依赖，另辟蹊径，锐意创新谋发展；必须坚持集聚优势、推进发展，既要发挥我市靠近珠三角、土地面积较大、海岸线长等固有优势，更要善于创造体制机制优势，打造粤东通往珠三角桥头堡，建设"东岸十市通衢"；必须坚持共建共享、激发活力，既要善于集中力量办发展大事，又要善于协调力量办民生实事，才能充分体现和发挥社会主义制度优越性，凝聚全体人民的智慧和力量谋发展；必须坚持创新机制、狠抓落实，带队伍创新，靠队伍落实，用全新的考核、督查、奖惩机制推动队伍提高公信力、执行力、免疫力，提高抓落实的水平。

　　在充分肯定成绩的同时，我们必须清醒地看到，我市发展还存在不少压力和困难：一是经济内推力不足。经济基础薄弱、产业链条短、工业增长拉力较弱、第三产业依然薄弱、创新不足的矛盾仍然十分突出。二是社会矛盾多发的难题尚未完全破解。进入工业化中期阶段，是社会矛盾凸显期，解决民生欠账、优化软环境是政府工作目前亟需解决的突出问题。三是全面"砍尾"后助力不强。已甩尾的大部分指标优势不大，全面 "砍尾"压力较大，"前移进位"推动力不足。四是干部执行力有待进一步提高。对此，我们必须高度重视，采取有效措施加以解决。

今后五年目标任务及2012年工作安排

　　今后五年，是我市加快发展的黄金机遇期，我们占有天时、地利、人和：省委、省政府赋予我市争当促进区域协调发展排头兵的历史重任，深汕合作上升为省级战略，转型升级战略全面实施，这些为我市加快发展提供了难得机遇；同时，我市发展内动力日益增强，区位优越、交通快捷、海岸线长、土地资源丰富等后发优势日益凸显，陆丰核电、华润海丰电厂、腾讯等一批重点项目相继落户，为我市加快发展提供了充足后劲，特别是当前全市上下推进科学跨越发展、建设幸福汕尾的热情高涨，为我市加快发展注入了强大动力。对汕尾的未来，我们充满信心、充满期待。我们要在市委的坚强领导下，团结带领全市人民，抢抓机遇，开拓创新，全力做好各项工作。

　　今后五年工作的指导思想是：高举中国特色社会主义伟大旗帜，以邓小平理论和"三个代表"重要思想为指导，深入贯彻落实科学发展观，全面实施"深汕合作、海岸经济、宜居城乡、人文环境"四大战略，着力打造"电力能源、电子信息、石油化工、现代旅游、现代物流、现代农业"六大基地，抓住黄金发展机遇，加大转变发展方式、推进区域协调力度，力图后来居上，全力建设宜居宜业宜憩的现代化滨海新城，为推动科学跨越发展、建设幸福汕尾，争当促进区域协调发展排头兵而努力奋斗。

　　今后五年的奋斗目标是：到2016年，地区生产总值达到1200亿元以上，年均增长17%；人均GDP达到4.1万元（约6300美元），年均增长16%；产业布局科学合理，产业结构明显优化，发展方式更加科学，经济社会发展更加协调。

　　2012年是新一届政府履行职责的开局之年，也是奠定未来五年发展基础至关重要的一年。主要目标任务是：地区生产总值增长16%；人均GDP增长15%；农业总产值增长6%；规模以上工业增加值增长33%；固定资产投资增长28%；社会消费品零售总额增长17%；外贸出口总额增长15%；外商直接投资增长10%，力争20%；地方财政一般预算收入增长25%；城镇居民人均可支配收入增长12%；农民人均纯收入增长12%；居民消费价格涨幅控制在4%左右；城镇登记失业率控制在3%以内；人口自然增长率控制在7&permil;以内；单位GDP能耗、主要污染物排放量完成省下达的指标。

　　实现今后一个时期的工作目标，要着力加快"八个转型"。

　　一、加快产业转型，构建现代产业体系

　　产业是经济发展的核心要素，加快产业转型是实现科学跨越发展的必然要求。要以工业园区、专业镇和海岸线为依托，按照项目带动的主体路径，致力建设电力能源、电子信息、石油化工、现代旅游、现代物流、现代农业六大产业基地，加快构建具有汕尾特色的海岸经济产业体系。

　　大力培育战略性新兴产业。培植发展电子信息、生物制药、节能环保、新能源、新材料、光电一体化等新兴产业。以深汕特别合作区和市城区为核心发展区，重点推进腾讯、芯灵电子、信利等项目建设，做大提升电子信息产业。加快融入珠三角东岸4+1（穗、深、惠、莞+汕尾）片区电子信息产业带，延长和完善电子信息产业链，努力打造高端新型电子信息产业集聚区。加快推进陆丰核电、华润海丰电厂等电力能源项目建设，全力打造电力能源基地。推进中油华城石化基地、粤东成品油管道汕尾段和天然气管道项目建设，发展精细化工及石化中下游产品加工等产业。充分利用高新技术，发展保健食品工程、药物工程，以及海产品综合利用和深加工技术。利用汕尾丰富的石英砂、高岭土、锆矿等矿产资源，发展新材料产业。

　　大力发展现代服务业。加快推进汕尾新港区规划建设，力争把汕尾新港区建成珠三角地区新的港口物流配套基地。加快建设市区水产品交易中心、陆丰内湖农副产品批发交易市场、汕尾铁路货运物流中心等一批专业市场和物流基地。强化旅游规划，优化旅游布局，做到谋定而后动，避免旅游资源占而不用，长期闲置；整合"红、蓝、绿、古、特"五色资源，构建不同风格的滨海、生态和城市景观区；培育旅游品牌，发展旅游产业，打造"珠东"现代旅游新城。加快建设集购物、休闲、餐饮和娱乐等功能为一体的核心商贸区，大力发展金融、信息等服务业，提升现代服务业发展水平。

　　大力推动传统产业改造升级。加强质量强市工作，运用现代信息技术、先进适用技术改造提升优势传统产业，壮大产业规模，力促产业链向微笑曲线两端延伸，突出抓好公平服装、可塘珠宝、碣石圣诞礼品、甲子家具配件、梅陇金银首饰工艺品、鹅埠制鞋、城东纺织和海产品深加工等专业生产基地建设，参与行业标准的制订，全力打造区域品牌，提高产品美誉度和产业竞争力。

　　大力抓好园区建设和招商引资。进一步调整园区布局和产业规划，完善园区政策，强化土地管理，加快园区基础设施和配套建设，突出产业特色，延长产业链条，全面提升工业园区的承载能力和招商选资水平。进一步完善我市省级开发区，力争每个县（市、区）建好一个工业园区。加强与珠三角地区在产业、基础设施、社会事业、政府服务、环保等方面的交流合作，促进生产要素合理流动。树立"大招商、招大商"理念，创新专业招商、产业招商、以商招商、以侨招商等方式，探索人才、管理、土地等要素招商。营造投资环境，落实优惠政策，重点加快研究制定合作区财政、税收、海关、金融、土地等招商引资优惠政策，以战略性新兴产业为导向，办好招商活动，大力引进世界500强企业、境外大型企业和中国500强企业、央企、民营大中型企业，做大招商引资增量。突出抓好深汕特别合作区招商引资工作，力争在高新技术产业、现代临港工业等产业招商中有新突破。创新进出口服务体系，推动贸易转型升级，做大做强外源型经济。

　　二、加快区域合作转型，以深汕特别合作区为引擎带动全市协调发展

　　深汕合作，事关全局。要坚持以"创新发展、先行先试"为核心，围绕"一年起好步，三年打基础，五年大发展"目标，努力创建全省乃至全国区域合作创新示范区。

　　突出创新体制。全面落实《深汕特别合作区基本框架方案》，完善合作区体制机制。按照精简高效原则，形成重大问题由深圳、汕尾两市高层决策领导小组协调解决，日常事务由合作区自主管理，合作区直接对省建立工作联系的机制管理架构，提高合作区行政效能。探索开发模式，积极引入有实力、有品牌效应的大型企业集团参与合作区的开发建设，吸引珠三角地区产业组团式转移入园，引进培育一批产值上百亿、大批产值上十亿的项目。组建合作区建设开发公司和土地开发投资公司，打造投融资平台，争取国家金融机构支持和省资金扶持。探索分期供地的土地供应模式，严格控制用地标准和项目准入门槛，加强已出让用地批后监管。

　　突出规划编制。立足科学发展，坚持经济发展和生态保护、民生改善并重，统筹编制合作区经济社会发展总体规划、土地利用规划、城市发展规划，努力实现合作区总体规划的统一融合。按照"总部＋基地"和"一区多园"的发展思路，主动与深圳和其他珠三角地区联动协调发展，合理安排合作区各类产业布局，大力提升产业和城市功能转移的承接服务能力。

　　发挥辐射作用。以合作区为引擎，带动全市县域经济全面协调发展。加快交通道路建设，以合作区鮜门火车站为基点，以合作区至市中心区为主轴，打造辐射全市的快速交通干道网。加大路网、站点、港区、仓储、产业配套等对接，拓展延伸合作区对全市的辐射。激发县域经济发展活力，努力把市城区建设成为高端产业创新区、宜居城市后起新秀；把海丰县建设成为转型升级先行区、县域经济排头兵；把陆丰市建设成为临港工业集聚区、跨越发展新兴市；把陆河县建设成为生态发展保障区、绿色崛起新山城；把红海湾开发区建设成为海岸经济发展区、运动休闲新乐土；把华侨管理区建设成为特色产业优化区、稳定发展新侨区。

　　三、加快城市转型，推进宜居宜业宜憩中心城区建设

　　有序扩大城市规模，深度优化公共空间，不断完善服务设施，全面提升城市品质，把汕尾建设成为宜居宜业宜憩的现代化滨海新城。

　　发挥规划引领作用。按照"绿色宜居、创业城市"要求，严格执行新修编的城市总体规划，全力建设汕尾新轴线，以汕尾中心城区为核心，以海丰、陆丰和合作区、陆河为次核，拓展城市规模，完善城市功能，提高城市控制性详细规划覆盖率。围绕打造"一（中）心一轴、一带三片（区）、多组团"的城市发展建设总体空间结构，在强化主城区建设的基础上北拓东进，突出"倚山、傍海、环湖、亲水、生态、休闲"的特色，营造"海在城中，城在山间"的城市空间形态特征。

　　加强城市基础设施建设。做好综合交通规划，立足大环境定位，突出市区建设、公共交通建设、港口建设，通过抓重点项目，加快立体交通、快速交通建设。大力推进厦深铁路汕尾段及火车站场配套设施建设，力争潮莞高速汕尾段全线开工建设。全力做好兴宁至汕尾高速公路及广州至汕尾、龙川至汕尾两条铁路的前期工作；加快市区凤飞山至捷胜公路、遮浪经大湖至上英公路、省道421线海丰圆山岭至品清路段改建工程、陆丰沿海公路碣石经湖东至甲子段等项目的建设。推进新湖大道、工业大道、红海西路、金鹏路等市区交通基础设施建设。加快玉台山公园、体育场、文化馆等设施建设。筹划好东涌、红草、马宫等镇（街道）的开发建设。完善一批"名医、名校、名居、名企、名区、名园"。引导房地产业健康有序发展。大力推进电网和信息化建设，打造光亮城乡和"智慧汕尾"。鼓励社会资本参与市政设施建设，推动城建投资主体多元化、资源利用市场化。

　　加强生态工程建设。按照要求规划好环境，保护好硬件设施。加快建设"千村千园"、"千里绿道"和"万亩荒山"绿化工程，大力打造城市景观带和沿海林业生态景观带。完善污水处理厂和城镇污水收集管网，推进市生活垃圾无害化处理中心建设。加大水环境综合治理，抓好河流两岸生态与景观建设，实行区域上下游联防联治。重点抓好奎山河整治和品清湖的综合治理及后续管理。

　　加强资源管理。加强对土地、海域、矿山等资源管理。坚持和落实最严格的耕地保护制度、最严格的节约集约用地制度，抓好土地总体规划的实施，促进土地资源的合理利用。发挥好市场在土地管理和调控中的作用，通过市场手段优化土地资源配置，依法垄断土地一级开发。以处置闲置土地为重点，盘活存量建设用地。加强和改进征地管理，完善征地安置措施，把更多的利益留给基层群众。加大"三旧"改造力度，因地制宜改造城中村，整治空心村。

　　加强城市管理。坚持精细管理，强化城市管理功能和责任，形成以市为指导、城区为主体、街道为重点、社区为基础、单位和居民参与的城市管理格局。创新社区管理模式，完善社区管理功能。强化市民家园意识，提高市民对城市的认同感和归属感。探索建立城市管理联席会议制度，调动职能部门共同参与城市建设管理的积极性。积极探索环卫事业市场化，加快环卫内部管理机制改革。加强城市交通管理，深入开展对违法违章建设的整治工作。

　　四、加快农村转型，千方百计提高农民收入

　　"三农"工作是建设幸福汕尾的着力点，提高农民收入是做好"三农"工作的落脚点。要加快农村转型发展，建设现代农业，激发农村经济发展活力，促进农民增收。

　　加强农业基础设施建设。加快"百里海堤爱海工程"238公里的海堤加固达标建设，重点建设好海丰东关联安围和陆丰"三甲"堤围。抓好百宗治洪治涝保安工程、农田水利工程和村村通自来水工程。抓好小型农田水利重点县和农田水利示范镇建设。保护好、利用好水资源，确保人民群众饮用水安全。组织实施好基本农田保护示范区、基本农田整治等现代标准农田建设项目，2012年力争全市整治农田5万亩以上。加快农业机械化进程，积极引导专业户运用大中型机械和设施农业机械。加强农产品质量安全体系建设，严格农产品标识管理和市场准入，确保农产品质量安全。加快社会主义新农村建设。制订名镇名村建设规划，稳步推进名镇名村示范村建设，抓好村庄整治，改善农村生产生活环境，提升农村宜居水平。

　　发展效益农业、特色农业。突出做好订单农业、科技农业、高效农业、品牌农业、安全农业等文章，实现农业转型升级。大力发展以无公害、绿色、有机食品为标准的原生态农产品，打造一批农业名牌产品。培育发展有地方特色的园艺优势产业，加快农业科技园区建设，完善 "十大菜篮子工程基地"、"十大无公害蔬菜生产基地"、"五大无公害原生态粮食生产基地"，以及市现代农业示范区、国泰五丰农产品加工示范区等现代农业园区。以我省建设国家海洋经济综合试验区为契机，加快现代水产养殖基地建设，发展深外海捕捞和水产品深加工业，推动水产龙头企业发展，抓好恒兴等大型水产项目实施，发展海洋经济支柱产业。加快汕尾中心渔港和遮浪渔港建设，带动全市其他渔港及其商贸配套设施建设，打造海产品物流商贸中心。

　　促进农村劳动力转移。积极开展农业生产技能培训，努力把广大农户培养成懂技术、善经营、会管理的现代农业生产者；深化与珠三角地区等地的合作，增强深汕特别合作区对全市劳动力的吸纳能力，建立完善劳务输出机制，实施一户一技能计划，拓宽就业渠道，继续推动渔农劳动力向非农产业转移，提高农村富余劳动力转移就业率，努力建立促进农民增收的长效机制。

　　加大扶贫开发力度。按照"选路子、筹票子、富脑子、盖房子、强班子"工作思路，增加财政投入，发动社会力量参与扶贫开发，加大对贫困村、贫困户的扶持力度；加强对非贫困村贫困户的扶贫工作，力促早日脱贫致富。

　　加强农村基层建设。加强镇级政权和执政能力建设，完善镇级管理体制，理顺责权利关系。稳步推进土地经营权有序流转，提升农业适度规模经营水平。认真实施村级公益事业一事一议财政奖补政策，加强农村集体资产管理，扎实推进农村综合改革，抓好基层社会管理体制改革试点，规范村务公开民主管理。强化政府在农村的服务职能，完善农民的诉求表达机制和矛盾调处机制，维护农村社会和谐稳定。

　　五、加快企业转型，夯实经济发展微观基础

　　企业是经济发展的微观基础。要牢固树立"善待企业"观念，像爱护自己的眼睛一样爱护企业家，推动企业转型升级。

　　扶持做大做强。全面落实扶持中小企业发展的政策措施，集中土地、劳动力、财力等资源要素向优势、骨干企业倾斜，鼓励企业科技创新、兼并重组和改制上市，加快培育一批主业突出、核心竞争力强的民营经济龙头企业，培植壮大财源税源，提高财税收入质量。大力扶持民营企业发展壮大。在市场准入、筹资融资、进出口贸易、税费征管、土地批租等方面，给予民营等非公经济更大优惠。鼓励企业在巩固国内市场的同时，开拓国际市场。

　　鼓励自主创新。加大科技投入，大力扶持以企业为主体的自主创新体系建设，引进项目研发中心、研究院所、产业孵化器，重点利用信利平板、"云计算"、芯灵电子、天洋太阳能光伏等高端技术项目带动研发基地落户，创建省级高新技术开发区。加强与中国科学院合作的"院地合作"联盟，深化产学研合作，逐步提升科技对经济发展的贡献水平。强化名牌带动，积极引导有条件的企业申报驰名商标、著名商标，以及国家、省名牌产品。

　　优化营商环境。进一步理顺政府与企业、市场、社会的关系，创新政府管理方式。依法规范政府投融资行为，加大中小企业融资力度。强化经济运行监测和调节，妥善应对各种经济风险。切实改善对市场主体的服务，继续完善"保姆式"、"一站式"、"订单式"等三种服务方式，增强企业投资和社会消费的信心，努力营造全社会尊重企业家、关心企业家、爱护企业家、支持企业家干事创业的良好环境。加强行业职业道德建设，引导企业家恪守道德底线，诚信守法经营、勇担社会责任，带动全社会形成健康规范的营商氛围，促进企业健康、持续、稳步发展。

　　六、加快体制机制转型，突出抓好重点领域和关键环节的改革

　　事实证明，越是欠发达的地区，体制机制的障碍越多。必须围绕破难题、增活力、上水平，突破传统思维模式和工作方式，深入推进重点领域和关键环节的改革。

　　抓好今年三大重点改革。落实好全省深化体制改革工作会议精神，大力培育发展和规范管理社会组织，改革登记管理体制，鼓励社会组织参与公共服务。探索创新保障房的规划建设机制、投入机制和分配管理制度，建立多层次住房保障体系。加大医疗保障制度改革创新力度，引入市场化管理方式改革基本医疗保险管理制度，健全基本医疗保险筹资和待遇水平调整制度，大力推进公立医院改革。

　　继续深化经济体制改革。积极探索深汕特别合作区体制机制改革，力求在开发模式、投资融资模式、土地供应模式和管理机制等方面有所创新；严格按照市场规律办事，进一步推进市场经济体制改革；加大力度抓好土地管理制度改革，合理发挥土地资源效益；推进财政体制改革，做大财政蛋糕；深化收入分配制度改革，切实提高劳动要素在初次分配中的比重。

　　继续深化行政管理体制改革。加快推进扩权强县、扩权强镇，增强县域经济自主发展活力；进一步转变政府职能，加快社会管理体制、事业单位分类、行政执法体制和综合政府服务体制改革，推进政府体制机制和管理方式创新。推进公务员分类管理，建立健全机构编制管理制度，探索实行政府雇员制。深化教育、科技、文化等其它社会事业改革。

　　七、加快社会服务转型，全力建设幸福汕尾

　　全面贯彻省委、省政府《关于加强社会建设的决定》，加快基本公共服务体系建设，推动社会事业繁荣发展，全面提高民生质量，让人民群众生活得更加幸福、更有尊严。

　　大力加强社会保障。抓好社保扩面征缴，完善社会保险制度，确保养老金等各项社保待遇按时足额发放；实施城镇居民养老保险，巩固和扩大新型农村养老保险；推进城乡居民医保一体化工作，逐步提高医疗保险待遇水平，做好社会保障卡发放工作，实现职工医保、居民医保、新农合"三网合一"。提高城乡困难群体补助标准。落实促进就业政策，多渠道开发就业岗位，强化职业技能培训和就业服务，重点抓好高校毕业生、农村劳动力、就业困难人员的就业工作。加强人文关怀，创建和谐劳动关系示范区。

　　大力发展教育事业。创建"教育强市"、"教育强县"、"教育强镇"。巩固提高高中阶段教育，加快调整中小学布局，实施中小学校舍安全工程。引进社会资金投入，鼓励和扶持社会力量办学。重点推进职业教育发展，加快市直中职学校、汕尾职院、理工职院、海丰职校和陆河职校建设。加快发展特殊教育，普及学前教育。继续实施"园区工程"和"名校、名校长、名教师"工程，把华师附中汕尾学校和陆河外国语学校建设成为区域性名校，积极推进陕西师大海丰附属学校和北师大珠海分校粤东附属学校建设。

　　大力发展卫生事业。加强医疗服务管理，加强卫生人才队伍建设，推进医德医风建设，全面提升医疗和公共卫生服务水平，促进基本公共卫生服务均等化。以创建三级甲等医院为目标，重点建设好市人民医院、逸挥基金医院和彭湃医院，形成良性竞争机制；逐步完善市直其他医疗卫生单位配套建设，加强县级医疗机构和乡镇卫生院建设，实现县有标准化综合医院、镇有规范化卫生院、村有合格卫生室，形成具有良好层级结构的城乡医疗服务体系；引进社会资金办卫生，推进医疗卫生事业健康可持续发展。

　　大力发展文化事业。以增强市民文明素质和文化软实力为目标，着力推进文化强市建设。实施好文化惠民工程，加快构建公共文化服务体系，提高文化产业在经济总量中的比重。加快推进中国（汕尾）民间文化艺术馆、市图书馆（扩建）和"数字影院"建设。推进基层文化设施全覆盖，实施"文化上网"工程。精心筹备汕尾市首届民间文化艺术节，深入开展群众广场文化活动。加强对国家级、省级文物保护单位、非遗名录项目的保护和开发利用。加强文艺精品创作和对外文化交流，做好文化人才培养和引进工作。

　　大力加强平安汕尾建设。加强社会治安综合治理，推进大平安体系建设。高度重视信访工作，建好人民来访接待厅、网上信访视频厅，切实解决群众反映强烈的突出问题。依法处置严重扰乱社会秩序的群体事件，抓好打黑除恶专项行动。继续加大打私工作力度，整顿和规范市场经济秩序，加强食品药品安全专项整治。健全完善安全生产长效机制，加大重点领域的安全专项整治力度，坚决遏制重特大事故发生。健全防灾减灾工作机制，增强防范和应对各类自然灾害的综合能力。进一步健全应急体系，提高应急保障能力。

　　大力推进其他社会事业发展。深入实施"全民健身计划"，抓紧筹建市体育中心。强基提质，大力推进人口和计划生育"升类创优"工程。加快殡葬改革步伐。抓好各类物资供应，控制物价过快上涨。实施妇女、儿童发展规划，关心支持老龄、慈善和残疾人事业，建设残疾人康复中心。深入开展国防动员、民兵预备役建设和"双拥"工作。发挥宗教界人士和信教群众在促进经济社会发展中的积极作用。统筹抓好统计、档案、保密、编制、方志、红十字、人防、对台、外事侨务、消防、气象、工会等工作，推动各项社会事业全面协调发展。

　　民生连着民心，民心凝聚民力。今年，我们将突出重点，加大投入，完善措施，办好人民群众普遍关心的十件惠民实事。一是投入6000万元建设以市区为重点的景观林带。二是开工建设市第三人民医院；市人民医院综合大楼投入使用。三是新增25万人领取养老金，确保全市城乡居民60周岁以上人员领取养老金，实现新型农村和城镇居民养老保险制度全覆盖。四是开工建设各类保障性住房1200套。五是6座已建污水处理厂全部投入运行，城镇污水处理率比2011年提高10%；投入1.5亿元建设市垃圾无害化处理中心。六是投入900万元建设完善200个村（社区）文化室。七是创建2个广东省教育强镇；扩建5所规范化乡镇中心幼儿园；新扩建4所特殊教育学校。八是新建市福利院、完善县级福利院。九是建设完善市政务服务中心，实现涉企审批事项100%进入中心；建设市应急指挥中心和市公共安全应急救援指挥中心。十是建设135公里农村硬底化公路；解决农村17.7万人饮水不安全问题。

　　八、加快政府转型，推进服务型政府建设

　　按照"为民、务实、清廉"的要求，进一步解放思想，转变观念，以改革创新的精神，加快政府转型，不断提高行政管理和服务水平，努力建设依法、高效、廉洁的服务型政府。

　　建设法治政府。自觉接受人大及其常委会的监督，坚持定期向市人大及其常委会报告重要工作；自觉接受政协民主监督；自觉接受媒体舆论监督；积极听取各民主党派、工商联、人民团体和社会各界的意见和建议，提高人大议案、政协提案的办理质量和效率。支持工会、共青团和妇联等人民团体更好地发挥作用。坚持依法行政，大力实施《汕尾市法治政府建设规划（2011-2015年）》，推进"六五"普法，严格按照法定权限和程序行使权力、履行职责，依法界定和规范政府在经济调节、市场监管、社会管理和公共服务等方面的职能。推进行政决策的科学化、民主化、制度化。健全公众参与、专家论证和政府决定相结合的行政决策机制，完善行政决策听证、专家论证、责任追究等制度。

　　建设高效政府。强化政府的社会管理和公共服务职能，切实解决涉及群众利益的难点、热点问题。坚持创新机制，进一步完善"责任、督查、奖罚"三大机制，力促工作落实，提高政府执行力。加快推进政务公开和电子政务建设，充分利用媒体、微博等信息手段做好政府信息公开工作；加快政务服务中心建设，提高办事效率；进一步办好市长热线，发挥市行政审批电子监察系统的作用，提升行政服务水平。加强公务员队伍管理，大力建设服务型机关。

　　建设廉洁政府。进一步加强对领导干部的廉政教育，认真落实反腐倡廉各项规定，促进政府廉政建设。深入开展机关作风建设，严格考核，兑现奖罚，强化行政监察、审计等专门监督，全面落实行政过错责任追究和领导干部问责制度，大力营造干事创业的良好氛围。

　　各位代表！回顾过去，我们无比自豪；展望未来，我们信心百倍！2012年是新一届政府履行职责的第一年，肩负的任务光荣而艰巨，让我们更加紧密地团结在以胡锦涛同志为总书记的党中央周围，在市委的坚强领导下，振奋精神，坚定信心，齐心协力，真抓实干，为推进科学跨越发展、建设幸福汕尾，争当促进区域协调发展排头兵而努力奋斗！
